# Aberrant inflammasome activation as a driving force of human autoimmune skin disease

**DOI:** 10.3389/fimmu.2023.1190388

**Published:** 2023-05-30

**Authors:** Tanja Fetter, Dennis Marinus de Graaf, Isabelle Claus, Joerg Wenzel

**Affiliations:** ^1^ Department of Dermatology and Allergy, University Hospital Bonn, Bonn, Germany; ^2^ Institute of Innate Immunity, University of Bonn, Bonn, Germany; ^3^ Institute of Human Genetics, University of Bonn, School of Medicine and University Hospital Bonn, Bonn, Germany

**Keywords:** inflammasome, keratinocytes, cutaneous lupus erythematosus, lichen planus, vitiligo, alopecia areata, bullous pemphigoid, psoriasis

## Abstract

Autoimmune skin diseases are understood as conditions in which the adaptive immune system with autoantigen-specific T cells and autoantibody-producing B cells reacting against self-tissues plays a crucial pathogenic role. However, there is increasing evidence that inflammasomes, which are large multiprotein complexes that were first described 20 years ago, contribute to autoimmune disease progression. The inflammasome and its contribution to the bioactivation of interleukins IL-1β and IL-18 play an essential role in combating foreign pathogens or tissue damage, but may also act as a pathogenic driver of myriad chronic inflammatory diseases when dysfunctionally regulated. Inflammasomes containing the NOD-like receptor family members NLRP1 and NLRP3 as well as the AIM2-like receptor family member AIM2 have been increasingly investigated in inflammatory skin conditions. In addition to autoinflammatory diseases, which are often associated with skin involvement, the aberrant activation of the inflammasome has also been implied in autoimmune diseases that can either affect the skin besides other organs such as systemic lupus erythematosus and systemic sclerosis or are isolated to the skin in humans. The latter include, among others, the T-cell mediated disorders vitiligo, alopecia areata, lichen planus and cutaneous lupus erythematosus as well as the autoantibody-driven blistering skin disease bullous pemphigoid. Some diseases are characterized by both autoinflammatory and autoimmune responses such as the chronic inflammatory skin disease psoriasis. Further insights into inflammasome dysregulation and associated pathways as well as their role in forming adaptive immune responses in human autoimmune skin pathology could potentially offer a new field of therapeutic options in the future.

## 1 Introduction

With its function as a barrier between the inner body and the environment, the skin plays a crucial role in the defence against pathogens (1). Keratinocytes, fibroblasts as well as skin resident immune cells express germline-encoded non-rearranged pattern recognition receptors (PRR), which sense molecular patterns of pathogens (PAMPs) as well as danger-associated molecular patterns (DAMPs) and homeostasis-altering molecular processes (HAMPs) from extracellular and subcellular compartments such as endosomes. These include Toll-like receptors (TLR), cytosolic receptors from the retinoic acid-inducible gene I (RIG-I)-like family (e.g. RIG-I and melanoma differentiation-associated protein 5), cyclic GMP-AMP synthase and stimulator of interferon genes, nucleotide oligomerization domain (NOD)-like receptor (NLRs), and Absent in melanoma 2 (AIM2)-like receptors (ALRs) such as AIM2 (2–7). Upon binding of a ligand to these receptors, inflammatory signaling pathways are activated, which lead to increased expression of cytokines and thus downstream activation of the adaptive immune system, often resulting in bystander tissue injury or cell death (8). Some of these PRRs are capable to assemble a large multiprotein signaling complex, the so-called “inflammasome”, first described by Martinon, Burns and Tschopp in 2002 (9). The inflammasome plays an essential role in combating foreign pathogens or tissue damage, but was also found to act as a pathogenic driver of metabolic and cardiovascular disorders (e.g. diabetes, atherosclerosis and gout), neurodegenerative diseases and autoimmune conditions (e.g. rheumatoid arthritis, systemic lupus erythematosus (SLE), systemic sclerosis (SSc) and Sjögren’s syndrome) when dysfunctionally regulated (10–13). Inflammasome formation can be triggered by pathogen- or host-derived stimuli, that emerge during tissue damage, infections or metabolic alterations (14). Following activation and assembly of the inflammasome, interleukins (IL)-1β and IL-18 are bioactivated, representing pivotal cytokines in connecting innate and adaptive immune responses, particularly by driving lymphocyte polarization. Activation of the inflammasome can also induce an inflammatory, lytic form of cell death known as pyroptosis (15).

The inflammasome is widely known for its pathogenic role in classical autoinflammatory syndromes with skin manifestations such as Muckle Wells syndrome, SAPHO (synovitis-acne-pustulosis-hyperostosis-osteitis)-syndrome, PAPA (pyogenic arthritis-pyoderma gangrenosum-acne)-syndrome and CANDLE (chronic atypical neutrophilic dermatosis with lipodystrophy and elevated temperature)-syndrome and familial mediterranean fever in which a dysregulation of the innate immune system has been described and which are often caused by mutations in NLR proteins (16). Recent studies suggest that inflammasome activation also contributes to autoimmune conditions of the skin including the T cell-dominant skin diseases vitiligo, alopecia areata (AA), lichen planus (LP) and cutaneous lupus erythematosus (CLE), the B-cell mediated autoimmune blistering disease bullous pemphigoid (BP) as well as the chronic inflammatory skin disease psoriasis, the latter which is characterized by a mixture of autoimmune and autoinflammatory responses. A better understanding of how dysregulated inflammasome activation contributes autoimmune skin disease may provide a rationale for targeting inflammasomes with immunotherapeutics for patients with these disorders.

In this review, we provide a brief overview of the basic components, function and activation of the inflammasome in the skin and as well as the physiological role of inflammasome forming proteins in the skin. We also present the current state of knowledge on inflammasomes in autoimmune skin diseases.

## 2 Basic components, activation and effector functions of inflammasomes with respect to the skin

In the skin, inflammasomes can be formed in keratinocytes, fibroblasts and also in skin associated immune cells such as macrophages, Langerhans cells and lymphocytes (17–21). Inflammasomes and associated proteins play a role both in the context of physiological functions such as keratinocyte differentiation and skin defence as well as pathological, inflammatory skin conditions (22–25). During cornification, intra- and extracellular processes occur such as proteolysis and nucleic acid degradation in order to establish a mechanical resistant cornified layer (26). These modifications, however, can lead to the generation of apparent danger signals such as endogenous nucleic acids, damaged organelles and altered ion concentrations by PRR and thus potentially entail activation of inflammasomes with release of IL-1β and pyroptosis. Therefore, differentiation is tightly controlled by anti-inflammatory mechanisms such as regulatory proteins, e.g. IL-37, IL-38, and IL-36RN, which can contribute to prevention of premature terminal differentiation (22, 27, 28). Autophagy is another important anti-inflammatory mechanism that occurs during cornification, as autophagosomes/lysosomes degrade intracellular contents and thus prevent inflammasome signaling, antigen presentation and preserve cellular homeostasis (29, 30).

Inflammasomes typically consist of a ligand sensor (e.g. NLR/ALR), an adaptor protein called apoptosis-associated speck-like protein containing a CARD (ASC), featuring a sensor-binding pyrin domain (PYD) as well as a caspase-binding caspase activation and recruitment domain (CARD), and proinflammatory caspase-1, referred to as the “canonical” inflammasome (9). The so-called “non-canonical” inflammasome is defined by direct activation of caspase-4 and caspase-5 in humans, occurring specifically as a result of sensing cytosolic lipopolysaccharide (LPS) from Gram-negative bacteria (31–34). Non-canonical caspases function as both sensor and effector molecules for LPS and do not require multiple protein components for ligand sensing, assembly and effector functions as is the case for canonical inflammasomes. Activated caspase-4 and caspase-5 can directly cleave gasdermin (GSDM) D, which is known to execute pyroptosis (32). The active, N-terminal cleavage product of GSDMD inserts into the cell membrane, induces cell swelling and lysis and is needed for the release of cytoplasmic contents into the extracellular space to promote inflammatory effector functions such as attraction of immune cells to the lesional site (32). Notably, caspase-4 and caspase-5 were also found to induce secondary activation of the canonical NLRP3 inflammasome in human monocytes (35).

**Figure 1:**
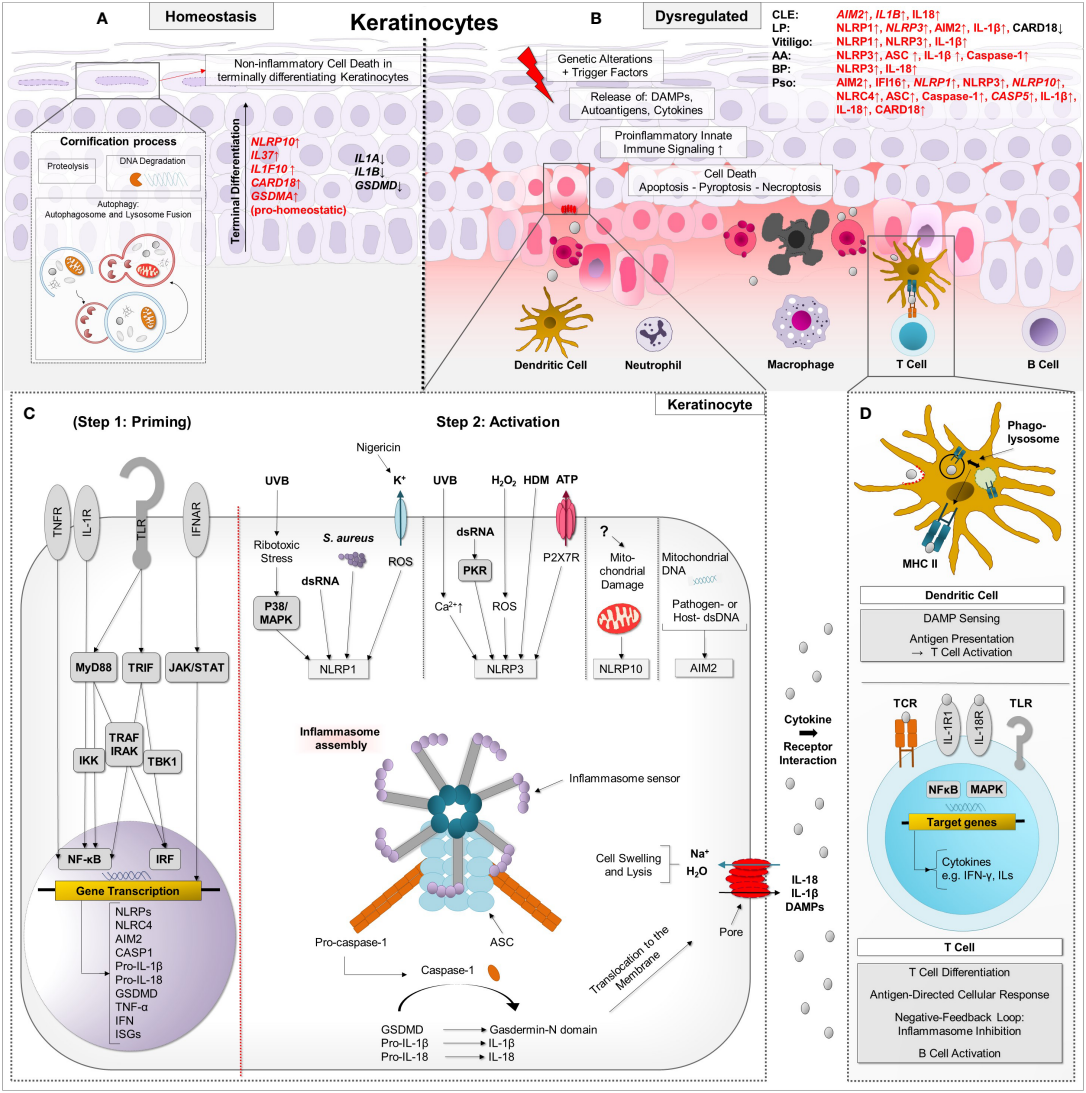
Simplified schematic presentation of the two-step regulatory process of inflammasome activation and proinflammatory downstream effects in the context of autoimmune skin diseases. **(A)** During physiological cornification, degradation of cellular components and proteolysis occur in keratinocytes, which have the capacity to activate innate immune pathways such as the proinflammatory inflammasome with subsequent pyroptosis. Specific regulatory mechanisms such as autophagy and anti-inflammatory proteins such as IL-37, IL-38 and CARD18 can keep pyroptosis under tight control. Under the course of terminal differentiation, mRNA of pro-homeostatic acting *NLRP10, IL37, IL1F10* and *GSDMA* is upregulated in keratinocytes, whereas mRNA of proinflammatory *IL1B*, *IL18* and *GSDMD* is decreased compared to basal epidermal keratinocytes. **(B)** In autoimmune skin diseases, terminal differentiation and inflammasome signaling can be dysregulated. Trigger factors lead to the release of DAMPs and inflammatory cytokines in keratinocytes, entailing innate immune signaling such as inflammasome activation. In a priming step, which is not mandatory for keratinocytes, TLR, TNFR, and IFNAR are activated by DAMP/PAMP and cytokines, entailing downstream NF-κB- and JAK-STAT signaling. Upregulation of inflammasome-associated proteins (such as NLRP, NLRC4, AIM2, pro-IL1β, pro-IL18) alongside others such as ISG leads to increased susceptibility to inflammasome stimuli, which can initiate inflammasome assembly in a second “activation” step. **(C)** In keratinocytes, NLRP3 inflammasome can be activated by UVB radiation followed by Ca^2+^ influx, dsDNA mediated by PKR, H_2_O_2_ *via* ROS production, house dust mites, also named Dermatophagoides, and P2X7R-activating ATP. NLRP1 inflammasome can be activated by UVB-induced ribotoxic stress *via* p38 signaling, dsRNA, *Staphylococcus (S.) aureus* and the K^+^ specific ionophore nigericin, which leads to ROS production. NLRP10 can be activated by distinct molecular events in damaged mitochondrial organelles, independent of mtDNA. AIM2 is known to recognize nucleic acids (extracellular, cytosolic DNA/RNA motifs and mtDNA). Upon inflammasome assembly, effector molecules IL-1β, IL-18 and GSDMD are activated. *Via* GSDMD-executed pyroptosis, DAMPs, interleukins and other cytokines are released through the pores. **(D)** Thus, these molecules can exert their effector functions on neighbouring cells, e.g. keratinocytes and dendritic cells. Inflammatory cells such as macrophages invade lesional skin and dendritic cells can activate T cells resulting in an antigen-driven cellular response. Keratinocytes can undergo further forms of cell death such as apoptosis and proinflammatory necroptosis. Caspase activation and recruitment domain (CARD), Chemokine (C-C motif) ligand (CCL), Chemokine (C-X-C motif) ligand (CXCL), Dendritic cell (DC), Neutrophil granulocyte (nG), Macrophage (Mph), Plasmacytoid dendritic cell (pDC), Toll-like receptors (TLR), Tumour necrosis factor receptors (TNFR), Nucleotide-binding oligomerization domain, Interferon-α/β receptors (IFNAR), Damage-associated molecular pattern (DAMP), Pathogen-associated molecular pattern (PAMP), Myeloid differentiation primary response 88 (MYD88), TIR-domain-containing adapter-inducing interferon-β (TRIF), IkappaB kinase (IKK), TNF receptor associated factor (TRAF), Interleukin-1 receptor-associated kinase 1 (IRAK), TANK-binding kinase 1 (TBK1), Caspase (Casp), NLR family CARD domain containing 4 (NLRC4), p38 mitogen-activated protein kinases (p38), A mitogen-activated protein kinase (MAPK), Staphylococcus aureus (S. aureus), double-stranded-RNA (dsRNA)-activated serine-threonine protein kinase (PKR), Hydrogen peroxide (H_2_O_2_), House dust mites (HDM), Adenosine triphosphate (ATP), P2X purinoceptor 7 (P2X7R), Mitochondrial DNA (mtDNA), Nuclear factor kappa-light-chain-enhancer of activated B cells (NF-κB), Janus kinase-signal transducer and activator of transcription (JAK-STAT), Leucine rich Repeat and Pyrin domain containing (NLRP), Absent in melanoma 2 (AIM2), Interleukin (IL), Interferon-stimulated genes (ISG), Reactive oxygen species (ROS), Pattern recognition receptor (PRR), Gasdermin A/D (GSDMA/GSDMD).

In general, activation of the canonical inflammasome is conducted in two steps (14). In the first step, PAMP/DAMP are sensed by a PRR or an IL-1R, which leads to a nuclear factor kappa-light-chain-enhancer of activated B cells (NF-κB)-dependent increase in expression of components of the inflammasome, as well as pro-IL-1β, which is mediated *via* NF-κB pathway. This step is understood as a “priming step” that positively influences the susceptibility of respective cells to inflammasome stimuli. Thus, this priming step also represents an important regulatory possibility at the transcriptional level. Notably, the expression of inflammasome components alone is not sufficient to form an inflammasome. The primed cell needs now to encounter an activating stimulus such as PAMP/DAMP *via* inflammasome sensors to induce inflammasome assembly. This is accomplished in a second step, named “activation step”: Following activation, the sensor assembles with ASC and the still inactive effector molecule pro-caspase-1 (14). The nucleated inflammasome may assemble into large helical fibrils that form a supramolecular signaling platform, upon which the executioner enzyme pro-caspase-1 can be bioactivated (36). Bioactive, caspase-1 is able to cleave pro-IL-1β and pro-IL-18 into their biologically active forms (37). IL-1β and IL-18 act as potent stimulators for other cells: signaling of IL-1β leads to activation of NF-κB-, MAPK-, and c-Jun N-terminal kinase-mediated signaling pathways and consequently to increased expression of proinflammatory cytokines such as IL-6 and tumor necrosis factor (TNF), recruitment of immune cells and development of specific T-cell and B-cell-driven inflammatory responses (38); IL-18 induces vascular inflammatory mediators such as the cell adhesion proteins intercellular adhesion molecule 1, vascular cell adhesion molecule 1 and E-selectin, and together with IL-12 induces IFN-γ production from T helper (Th) cells (39) and generally stimulates both Th 1 and Th 2 responses (40). Caspase-1 also cleaves GSDMD, which oligomerizes and inserts into cell membranes to form a pore and executes pyroptosis (41, 42). IL-1β and IL-18 exit the cell through these pores, along with other alarmins such as high mobility group box 1 protein (HMGB1) and IL-1α, and are thus enabled to exert their effector functions on surrounding (immune) cells, which is another important function of GSDMD activation (**Figure 1**) (43). Notably, in human monocytes, a so-called “alternative” inflammasome has been described, in which the NLRP3 inflammasome triggered by LPS leads to the secretion of IL-1β, but is independent of GSDMD and thus does not induce pyroptosis (44).

NLRs typically consist of a central NACHT domain, a C-terminal series of leucine-rich repeats (LRRs) for ligand sensing and an N-terminal homotypic protein–protein interaction “effector” domain such as PYD or CARD. They are divided into five subfamilies (NLRA, NLRB, NLRC, NLRP and NLRX) based on the type of N-terminal domain. Some of them are known to nucleate an inflammasome such as NLRC (with C for CARD) and NLRP (with P for PYD), the latter including the most studied sensor family (45). Of note, NLRP10, which was recently discovered to form an inflammasome complex, lacks a full LRR domain, implying another mechanism of ligand sensing (46). Another group represents the Pyrin and hematopoietic interferon-inducible nuclear (HIN) domain family members AIM2 (47) and IFN-γ-inducible protein 16 (IFI16) (48). AIM2 contains a PYD and a HIN domain, of which the latter serves as ligand sensor (49). AIM2 sensor protein was found to be strongly expressed in the entire epidermis in healthy human skin in one study (5), in contrast to another study, in which it was weakly detectable in normal human skin (23). NLRP1 and NLRP3 sensor proteins were found only weakly expressed in the basal epidermal layer of human healthy skin (19).

Dermal fibroblasts were also found to express inflammasome sensors, such as AIM2, as well as caspase-1 and IL-1β in response to arboviruses. Accordingly, silencing of caspase-1 was found to enhance replication of the arbovirus family member Chikungunya virus in dermal fibroblasts (50). In another study, cultured dermal fibroblasts were found to secrete high amounts of IL-18 as a response to NLRP3 inflammasome activation by UV-induced advanced glycation end product accumulation (20). In cultured dermal fibroblasts of SSc patients, *AIM2* and *NLRP3* mRNA expression was increased compared to normal fibroblasts (13). Transcripts of *IL18* and *IL1B* were also found to be increased and correlated with elevated protein secretion of respective interleukins by SSc fibroblasts (13).

## 3 Ligand sensing and inflammasome activation in keratinocytes

Keratinocytes are known to express several inflammasome sensors such as NLRP3 (51), NLRP1 (52), AIM2 (5), NLRP10 (25) and NLRC4 (53) as shown in **Table 1**. In principle, keratinocytes constitutively express mRNA and protein IL-1α, IL1-β, IL-18, ASC and pro-caspase-1 and are basically equipped with all inflammasome components (37, 55–58). They were also found to constitutively express *CASP4* mRNA (56) as well as caspase-5 mRNA and protein (55). Of note, mRNA expression levels of *IL1B, CASP1* and pyroptosis execution protein *GSDMD* are downregulated during terminal differentiation of human keratinocytes *in vitro*, and mRNA of *NLRP10* – besides mRNA of the anti-inflammatory cytokines *IL37* and *IL1F10* - was found to be strongly increased in differentiated keratinocytes (27).

**Table 1:** Expression of inflammasome-forming ligand sensors in different cell types of the skin.

Cell Type	Ligand sensors	References
Keratinocytes	NLRP1 NLRP3 NLRP10 NLRC4 AIM2	(5, 25, 51, 53, 54)
Fibroblasts	NLRP3 AIM2	(20, 50)
Langerhans cells	NLRP1	(21)
Melanocytes	–	–

Indeed, keratinocytes are capable of forming inflammasomes as shown for UV-stimulated keratinocytes forming ASC specks (59) and, importantly, can initiate inflammatory signals without prior priming (37). In keratinocytes, AIM2 can sense both host derived or viral cytosolic and mitochondrial (mt)DNA reacting with a strong IL–1β release (5, 60–63). Interestingly, AIM2 protein was also found to be upregulated in cultured human keratinocytes upon IFN-γ stimulation, which represents an important mediator of Th1 based adaptive immune responses (23, 64). NLRP10 inflammasome can be activated by hitherto unknown molecular events associated with damaged mitochondrial organelles, independent of mtDNA (25). NLRP1 senses UV radiation (65), ribotoxic stress (66, 67) double-stranded RNA (68) and skin colonization with *Staphylococcus aureus* (69) in keratinocytes, of which the three latter were discovered only recently. NLRP3 inflammasome is best characterized so far and generally recognizes fungal, viral, and bacterial motifs as well as particulates and crystals (14, 70). In keratinocytes, NLRP3 was also found to be triggered by UVB radiation at increased cytoplasmatic calcium (Ca^2+^) concentrations (37). Furthermore, UVB exposed keratinocytes can secrete IL-1β as a response to NLRP3 inflammasome activation without a specific priming signal (37, 71). Caspase-4 was also found to be secreted by keratinocytes upon UVB radiation (72). However, *in vitro* studies on human primary keratinocytes indicate that UVB sensing predominantly leads to activation of NLRP1 inflammasome (65). Moreover, the assembly of NLRP1 inflammasome is initiated as a response to UVB-induced ribotoxic stress, mediated *via* p38/mitogen-activated protein kinase (MAPK) signaling (65, 66). Interestingly, pathogenic germline variants in the *NLRP1* gene have been identified in patients with two phenotypically overlapping skin diseases, namely multiple self-healing palmoplantar carcinoma (MSPC) and familial keratosis lichenoides chronica (FKLC). These gain-of-function variants lead to constant spontaneous inflammasome activation in keratinocytes and increased paracrine IL-1 signaling, leading to skin inflammation and epidermal hyperplasia (73).

In addition to UV radiation, in keratinocytes, NLRP3 can also be activated by dsRNA *via* the double-stranded RNA-activated protein kinase (PKR) (51), extracellular adenosine triphosphate (ATP) which binds to the P2X7 receptor (74), H_2_O_2_ *via* reactive oxygen species (ROS) (75), the house dust mite (76) and increasing Ca^2+^ concentrations (37, 75). Of note, in other cell types, the NLRP3 inflammasome was found to get activated by other molecular signatures indicating cellular damage such as osmotic pressure and cell swelling, potassium efflux and lysosomal damage (14, 77).

These intracellular conditions can also be considered as positive regulatory mechanisms of inflammasome activation. In general, inflammasome activation is tightly regulated under physiological conditions and often altered in inflammatory skin conditions. Several mechanisms are known to contribute to inflammasome regulation such as posttranscriptional or posttranslational modifications (e.g. microRNA and long non-coding RNA as well as phosphorylation and deubiquitination, respectively) and molecules directly or indirectly interacting with different components of inflammasomes or associated proteins such as CARD-only proteins (COP) or PYD-only proteins (POP) to prevent exceeding inflammatory responses. Regulatory mechanisms of inflammasome assembly are discussed in detail elsewhere (14, 36, 70).

Exposure of keratinocytes to effector cytokine IL-18 *in vitro* leads to upregulation of major histocompatibility complex (MHC) II and production of the CXCR3+ cell attracting chemokine CXCL10 (78). Moreover, in keratinocytes, pyroptosis was found to be executed mainly by other GSDM family members than GSDMD such as GSDMA and GSDME. The protease virulence factor SpeB of human pathogen *group A Streptococcus* induces pyroptosis in keratinocytes *via* GSDMA (79, 80). In another study, UVB radiation was reported to induce GSDME-mediated pyroptosis in keratinocytes (81). Only high doses of UVB radiation were found to induce pyroptosis *via* GSDMD (82). Collectively, GSDMA, which is strongly expressed in differentiated human keratinocytes (27), is considered the predominant GSDM family member in the skin (79).

## 4 Role of inflammasomes in autoimmune skin diseases

In both T-cell and B-cell mediated autoimmune skin diseases several inflammasomes are known to contribute to the activation of a pathological adaptive immune response. There is a spectrum of inflammatory skin diseases that are characterized by strong upregulation of IFN and recruitment of effector T cells (83, 84). This spectrum includes, but is not limited to, CLE (85), LP (86, 87), vitiligo (88) and AA (89–91), which are considered autoimmune diseases (92–94). These diseases come along with a predisposing genetic background, particularly gene polymorphisms of components of the innate and adaptive immune system. These genetic alterations can result in increased proinflammatory responsiveness of innate immune pathways, increased antigen presentation by HLA molecules and other dysfunctional mechanisms such as impaired nucleic acid clearance and autophagy (95). Upon exposure to triggering factors such as UV radiation, infections and drugs, epithelial cells in the skin can respond with cellular stress and the release of cytokines and cellular components such as endogenous nucleic acids that reflect DAMPs and potential autoantigens. DAMPs can be recognized by surrounding antigen-presenting cells (APCs) and other neighboring cells *via* PRRs. APCs induce maturation and clonal expansion of autoantigen-specific B and T lymphocytes. Upon repeated autoantigen contact, activated B cells can differentiate into plasma cells to produce specific autoantibodies against nuclear components and T cells can migrate into lesional tissues, attracted by released cytokines such as CXCL chemokines, to support B cell activation and exert cytotoxic effects against epithelial cells, resulting in further release of DAMPs (95–98). Effector T cells specifically target basal keratinocytes in CLE (85) and LP (86), melanocytes in vitiligo (99) and follicular epithelial cells driving the loss of hair follicle immune privilege in AA (100).

These disorders are driven by self-reactive T cells whereas other cutaneous autoimmune diseases are characterized by a strong B-cell- and autoantibody signature such as the most common autoimmune blistering skin disorder BP (101–103). BP is driven by autoantibody-mediated indirect destruction of the hemidesmosomal proteins collagen XVII (BP180) and dystonin-e (BP230) in the skin and mucosa. The respective autoantibodies induce complement activation and attraction of leukocytes and mast cells, which release proteases that destroy the dermoepidermal adhesion and drive inflammatory cytolysis (101, 102). This destruction of dermoepidermal adhesion leads clinically to tense blisters, which may also occur after a non-blistering phase of eczematous or urticarial skin lesions (101, 102). Since autoantibodies were found sufficient to cause loss of dermoepidermal integrity, autoreactive B cells are considered crucial players in the pathogenesis of BP (102).

Psoriasis is a chronic inflammatory skin disease with a broad spectrum of skin manifestations and underlying pathogenetic mechanisms. In the most common form, plaque psoriasis, the autoimmune response in the form of proinflammatory Th1 and Th17 cells and the IL-17/IL-23 immune axis particularly drives pathogenesis. In a rarer form, pustular psoriasis, autoinflammatory responses are dominating, particularly IL-1 and IL-36 cytokine signaling (104). A detailed description of pathogenic mechanisms can be found elsewhere (104, 105). Briefly, in autoimmune-dominated forms of psoriasis, cellular components such as endogenous nucleic acids and antimicrobial peptides, e.g. the cathelicidin LL37, which are released by stimulated keratinocytes, are considered significant in the initiation of skin lesions. Nucleic acids can form complexes with LL37, which is overexpressed by psoriatic lesional keratinocytes in comparison to healthy skin (106). These complexes can be sensed by plasmacytoid dendritic cells (pDCs), entailing increased IFN-α production of the respective cells (107). The release of IFN-α fosters maturation and activation of resident dermal dendritic cells, which produce the proinflammatory cytokines IL-23 and IL-12 and present cellular components as autoantigens to naïve T cells in the draining lymph nodes, thus inducing differentiation of Th1 and Th17 cells (108). Both Th1 and Th17 cells are known to release IFN-γ besides other inflammatory cytokines such as TNF-α, IL-17 and IL-22, which leads to the production of neutrophil-recruiting proinflammatory CCL and CXCL chemokines in keratinocytes. IL-1β in combination with IL-23 and IL-6 was found to induce IL-17 production in naïve T cells, thereby promoting T cell differentiation (109). Moreover, IL-1β also induced expression of the effector cytokine IL-17 in Th17 cells *in vitro*, thus driving lesional inflammation (110). These results suggest that inflammasome effector functions are directly linked to T-cell-driven autoimmunity in psoriasis pathogenesis.

Next, we review what is known to date about the role of inflammasomes in autoimmune skin diseases, of which the summary can be found in **Table 2**.

**Table 2:** Summary of main findings on inflammasome-associated proteins and pathways in different cutaneous autoimmune diseases.

CAD	Findings on inflammasomes and associated proteins	References
**Cutaneous lupus erythematosus (CLE)**	**Genetic alterations increasing susceptibility to LE:** • SNPs in *IL1B, NLRP1 and NLRP3*	(111–115)
	**CLE skin lesions (compared to skin of healthy controls):** • Increased *AIM2* mRNA expression • Increased IL-1β mRNA and protein expression • Increased IL-18 protein expression	(116–119)
	**Other (*ex vivo* experimental) findings**: *Ex vivo* cultured keratinocytes of CLE patients: • Increased concentrations of IL-18 protein compared to healthy cultured controls • Stimulation of CLE keratinocytes with IL-18 leads to increased CXCL10 expression compared to unstimulated cells	(116)
**Lichen planus (LP)**	**Genetic alterations increasing susceptibility to LP:** • SNPs in *IL18* in patients with oral LP	(120)
	**LP skin lesions (compared to skin of healthy controls)**: • Increased NLRP1, AIM2 and IL-1β protein expression • Increased *AIM2* and *NLRP3* mRNA expression • Decreased pro-IL-1β mRNA expression • Decreased protein expression of CARD18	(121–123)
	**Oral lichen planus lesions (compared to tissue of healthy controls):** • Increased protein expression of NLRP3, ASC, caspase-1 and IL-1β in oral epithelia and connective tissues	(124)
**Vitiligo**	**Genetic alterations increasing susceptibility to vitiligo** • SNPs in *NLRP1* significantly associated with generalized vitiligo	(125–129)
	**Vitiligo skin lesions (compared to skin of healthy controls):** • Increased NLRP1 protein expression in perilesional skin of patients with progressive disease, particularly in the entire epidermis, also compared to lesional skin of vitiligo patients • In contrast to finding above: Increased NLRP1 protein expression restricted to Langerhans cells in leading edge vitiligo skin lesions • Increased NLRP3 and IL-1β protein and mRNA expression in perilesional skin of patients with progressive disease, also compared to patients with stable vitiligo disease	(21, 75, 130)
	**Serum of vitiligo patients (compared to healthy controls):** • Increased concentration of IL-1β in patients with progressive disease, also compared to patients with stable disease	(75)
	**Other (*ex vivo* experimental) findings:** *Ex vivo* cultured peripheral blood CD4+ and CD8+ T cells from vitiligo patients: • Increased proinflammatory cytokine expression after stimulation with supernatant of H_2_O_2_ treated cultured keratinocytes and abrogation of cytokine expression by *NLRP3* knockdown in respective keratinocytes	(75)
**Alopecia areata (AA)**	**Genetic alterations increasing the susceptibility to AA**: • Polymorphisms in *IL18*, *IL1B* and *IL1RN*	(131–134)
	**AA scalp skin lesions (compared to scalp skin of healthy controls):** • Increased *IL1B* mRNA expression in scalp biopsies of patients • Increased NLRP3, ASC, caspase-1 and IL-1β protein expression in lesional skin, particularly in the outer root sheath (ORS) of hair follicles, of patients	(135–138)
	**Serum of AA patients (compared to serum of healthy controls):** • Increased concentration of IL-18 in patients with extensive disease	(139)
	**Other (*ex vivo* experimental) findings:** Effects of stimulation of *ex vivo* cultured ORS cells from AA patients with poly(I:C) compared to unstimulated cultured ORS cells: • Increased *NLRP3, ASC*, *CASP1* and *IL1B* mRNA expression • Increased NLRP3, pro-caspase-1 and pro-IL-1β protein expression • Increased secretion of active capsase-1 and IL-1β • Increased co-localization of NLRP3 and ASC	(138)
**Bullous pemphigoid (BP)**	**BP skin lesions (compared to skin of healthy controls):** • Increased IL-18 protein expression in lesional skin • Increased NLRP3 protein expression in lesional skin	(18, 140)
	**Serum and blister fluid of BP Patients (compared to healthy controls):** • Increased concentration of IL-18 in both serum and blister fluid of BP patients • Positive correlation of IL-18 concentrations with anti-BP180-NC16A autoantibody titers in serum of BP patients	(140)
	**PBMCs from BP patients (compared to healthy controls)**: • Increased *NLPR3, ASC*, *CASP1* and *IL18* mRNA expression • Increased NLRP3, ASC, (pro-)caspase-1 and pro-IL-18 protein expression	(140)
**Psoriasis**	**Genetic alterations increasing susceptibility to psoriasis** • SNPs in *NLRP1*, *NLRP3*, *AIM2*, *CARD8* and *CARD14*	(141–147)
	**Psoriasis skin lesions (compared to skin of healthy controls):** • Increased *AIM2*, *IFI16*, *NLRP3*, *NLRP10*, *PYCARD*, *CASP1, CASP5*, *IL1B*, *IL18* and *CARD18* mRNA expression in lesional skin • Increased NLRP3, NLRC4, IFI16, AIM2, ASC, caspase-1, inactive caspase-5, IL-1β and IL-18 protein expression in lesional skin • Increased *NLRP1*, *NLRP3*, *AIM2*, *PYCARD* and *CASP1* mRNA expression and CARD18 protein expression in lesional as well as non-lesional skin • DNA fragments detectable in the cytosol of keratinocytes from psoriatic skin lesions	(23, 53, 64, 148–153)
	**Peripheral blood of psoriasis patients (compared to healthy controls):** • Increased ASC, IL-1β and IL-18 levels in untreated patients • Increased NLRP1 and NLRP3 protein expression in peripheral blood CD4+ lymphocytes, CD14+ monocytes and CD16+ neutrophils • Increased AIM2 protein expression in CD14+ monocytes and CD16+ neutrophils	(154, 155)
	**Other *(ex vivo)* experimental findings:** Cultured keratinocytes: • Increased *AIM2* and *CASP5* mRNA as well as AIM2 protein as a response to IFN-γ • Increased IL-1β release and secretion of bioactive caspase-1 following pretreatment with IFN-γ and TNF-α and stimulation with poly(dA:dT) • Inhibition of IL-1β release as a response to exposure of LL37-DNA complexes • Decreased *AIM2* and *CASP1* mRNA expression and increased IL-1β protein expression following silencing of *CARD18* in IFN-γ- and TNF-α-pretreated and poly(dA:dT)-stimulated keratinocytes • Increased *NLRC4* mRNA expression *via* different stimuli: (i) keratinocyte confluence, (ii) air exposure, (iii) UV radiation, (iv) increased Ca^2+^ and (v) oxidative stress from H_2_O_2_ exposure • Increased *NLRP3*, *pro-IL-1β* and *pro-IL-18* mRNA expression in whole blood after exposure to TNF-α • Increased caspase-1 activity following TNF-α-stimulation of CD14+ and CD16+ peripheral blood cells • Downregulated *pro-IL-1β* mRNA expression following treatment with anti-TNF-α antibodies in whole blood	(23, 53, 64, 152–154)

### 4.1 Lupus erythematosus

Lupus erythematosus (LE) is a chronic autoreactive B- and T-cell-mediated inflammatory disease that can be isolated to the skin or present with systemic manifestations affecting multiple organs, joints and vessels, featuring skin lesions in approximately 70% of cases (94). The first evidence for a possible involvement of the inflammasome in LE was reported 2004 by Calvani et al., who described that concentration of IL-18 was elevated in the serum of SLE patients compared to healthy controls (111). In addition, a certain range of single nucleotide polymorphisms (SNPs) in inflammasome associated genes such as *IL1B* (rs1143629) (112), *NLRP1* (rs2670660) (113) and *NLRP3* (rs10754558) (114, 115) has been associated to an increased susceptibility to LE in affected patients compared to healthy controls in a number of studies investigating different ethnic subgroups each. Moreover, the *NLRP3* polymorphism rs10754558 also positively correlated with LE disease severity (115). In LE skin lesions and *ex vivo* cultured keratinocytes of CLE patients the effector cytokine IL-18 was found increased compared to healthy controls. Keratinocytes cultured with IL-18 were found to express high amounts of the chemokine CXCL10 when compared to unstimulated cells, which attracted co-cultured T cells (78, 116). In addition, *AIM2* mRNA was found highly expressed in CLE skin lesions compared to healthy skin (117, 118). Notably, UV radiation represents an important environmental disease driver in CLE patients and is a well-known direct activator of the inflammasome (156, 157). In one study, only interleukin-1β protein and mRNA expression was significantly upregulated in the lesional skin of CLE patients compared to their uninvolved skin. Relevantly, NLRP1, NLRP3, ASC and caspase-1 protein expression were not significantly upregulated in CLE skin lesions compared to healthy controls. (119). This suggests that not all inflammasome proteins contribute equal to pathogenesis, though likely only in certain CLE subtypes or molecular profiles.

### 4.2 Lichen planus

LP is considered a type I IFN-driven, T-cell mediated disease affecting the skin, mucous membranes and nails. LP skin lesions typically feature polygonal, flat, purplish papules or plaques with an overlying, reticular, white scaling called Wickham’s striae (158). LP lesions can vary in size and occur either localized, particularly on the extremities, or rarely disseminated (159). As for CLE, the IFN-CXCL10-CXCR3+ axis plays a crucial pathogenetic role, leading to T cell-induced necroptosis of basal keratinocytes reflecting the typical histopathologic pattern of interface dermatitis (86, 87). LP is also characterized by premature terminal differentiation, which refers to early development of a focal thick granular layer and a compact orthokeratotic stratum corneum (160). So far, there is only one study which investigated the association of four common genetic variants in inflammasome-associated genes to LP: SNPs in *IL18* were both found to be associated with an increased susceptibility to oral lichen planus (OLP) (rs1946518) as well as OLP severity (rs187238) in a cohort of Chinese patients (120).

In lesional skin of LP patients, IL–1β, AIM2 and NLRP1 protein were found to be highly expressed compared to skin of healthy controls (121). AIM2 protein was expressed both in the dermis and epidermis whereas NLRP1 and IL-1β were only found significantly increased in the dermis in skin lesions of LP patients (121). LP skin lesion samples showed higher *AIM2* (121) and *NLRP3* (122) transcript levels whereas *NLRP1* transcript levels were similar compared to healthy controls (121). Interestingly, in the same study, *IL1B* mRNA expression was found to be significantly downregulated in LP skin lesions compared to healthy controls (121), which may be due to a regulatory feedback mechanism leading to reduced inflammasome priming.

In addition, whereas TLR4 stimulation had no influence on IL-1β protein expression in PBMCs of LP patients compared to healthy controls, TLR7/8 stimulation was found to decrease IL-1β protein expression in PBMCs of LP patients (121). TLR activation is thought to serve as a priming signal for inflammasome activation. TLR4 and TLR7/8 can activate both NF-κB and IFN regulatory factor (IRF) pathways (via MyD88 or TIR-domain-containing adapter-inducing interferon-β) and thus induce an inflammatory response, e.g. cytokine and chemokine expression, or an antiviral state *via* type I IFN expression, respectively (161). The authors suggested that IRF related to type I IFN may inhibit inflammasome activation.

As for CLE, nucleic acids are proposed to play an immunostimulatory role in LP pathogenesis and represent a potential trigger factor for inflammasome priming and activation (162). In OLP, the concentration of cell-free DNA, which can be released by damaged cells, was found to be significantly increased in the saliva and plasma of OLP patients compared to those of healthy controls and positively correlated with the amount of inflammatory cytokines and clinical features (163). Protein expression of NLRP3, ASC, caspase‐1 and IL‐1β was also found increased significantly in oral epithelia and connective tissues of OLP patients compared to tissue of control subjects (124). In the same study, protein expression of these respective inflammasome components positively correlated with NF-κB signaling molecule protein expression and activity-enhancing O-GlcNAcylation in OLP patients compared to control subjects.

In another study, the protein expression of the regulatory protein and COP family member CARD18 was significantly downregulated in LP skin lesions compared to healthy controls (123). CARD18 is known to prevent inflammasome effector functions by inhibiting the generation of active IL-1β and IL-18 through direct interaction with pro-caspase-1 (164, 165) and was found to be highly expressed on both mRNA and protein level in differentiated keratinocytes under physiological conditions (123). These findings may be of particular interest, as LP is considered a disorder characterized by a premature terminal differentiation of keratinocytes which may be accelerated by hyperactive inflammasome activation and pyroptosis, potentially supported by the lack of regulatory CARD18.

### 4.3 Vitiligo

The autoimmune skin disease vitiligo is characterized with patchy depigmentation of the skin, overlying hair and oral mucosa that can manifest either locally or generalized (166). Depigmentation results of cytotoxic effector T cells directed against melanocytes (167). IFN-γ and Janus kinase/signal transducers and activators of transcription-mediated CXCL chemokines such as CXCL9 and CXCL10 are important proinflammatory messengers in initiation and progression of the disease (88, 99, 167, 168). The release of ROS triggered by both exogenous and endogenous stimuli such as UV-radiation, monobenzone and melanin itself is known to be crucial for the inflammatory response, inducing DNA damage, protein oxidation and fragmentation, lipid peroxidation and thus damage of cell organelles (167). Stressed melanocytes are also known to release exosomes containing microRNA, melanocyte specific antigens, heat shock proteins and other DAMPs, which can be recognized by NLRP1 and NLRP3 (167, 169, 170). Genetic polymorphisms are considered to account for 80% of vitiligo risk (167). Regarding the inflammasome, several polymorphisms in *NLRP1* on chromosome 17 were found to be associated with the occurrence of vitiligo (125–128). In the *NLRP1* extended promotor region, the SNPs rs2670660 and rs1008588 showed a significant association with generalized vitiligo in an Arabic cohort (128). This in line with findings in a Romanian cohort, in which rs2670660 showed the most significant association with generalized vitiligo (126). The SNP rs2670660 was also found significantly associated with generalized vitiligo (besides two other SNPs rs69502867 and rs8182352) in USA and UK families (125). Other reported SNPs significantly associated with generalized vitiligo in USA und UK families, which included rs6960920 and rs734930 on chromosome 7 and rs4744411 on chromosome 9 and for which the authors furthermore report an interaction with a SNP in *NLRP1* (129). NLRP1 and IL-1β protein expression was strongly positive in perilesional skin of vitiligo patients with disease progression, particularly in the entire epidermis, compared to skin of healthy controls. Remarkably, the expression of NLRP1 and IL-1β was almost absent in lesional skin of vitiligo patients, similar to that in the skin of healthy control subjects (130). In another study, higher expression of NLRP1 protein in leading edge vitiligo skin biopsies compared to healthy control skin were found, however, NLRP1 was almost exclusively detected in Langerhans cells (21).

Regarding the NLRP3 inflammasome, both protein and mRNA concentrations of NLRP3 and IL-1β were found increased in perilesional epidermal samples of vitiligo patients with progressive disease compared to healthy controls and vitiligo patients with stable disease (75). Moreover, IL-1β serum concentrations are increased in vitiligo patients with progressive disease compared to vitiligo patients with stable disease and healthy controls and positively correlated with the severity of disease progression (75). However, other inflammasome components including ASC, caspase-1 and effector cytokine IL-18 mRNA or protein were comparable between perilesional epidermal samples from vitiligo patients at any stage and healthy control samples (75). In cultured primary normal human epidermal keratinocytes (NHEK) and HaCaT keratinocytes, H_2_O_2_-induced mitochondrial ROS production led to colocalization of NLRP3 and ASC as well as upregulation of caspase-1 activity and IL-1β secretion, thus indicating inflammasome assembly. This is supported by the finding that knockdown of NLRP3 in HaCaT attenuated H_2_O_2_-induced caspase-1 activation and IL-1β secretion (75). In this study, the authors also found that transient receptor potential cation channel subfamily M member 2 (TRPM2), which is known to induce Ca^2+^ influx into melanocytes and keratinocytes under oxidative stress, acts as a mediator of NLRP3 inflammasome activation: TRPM2 was highly expressed in H_2_O_2_-stimulated cultured keratinocytes and knockdown of TRPM2 inhibited caspase-1 activity, IL-1β secretion and impaired colocalization of ASC and NLRP3 in the respective cultured keratinocytes (75). The authors further investigated the response of *ex vivo* cultured peripheral blood CD4+ and CD8+T cells from vitiligo patients to supernatant of H_2_O_2_-treated HaCaT. CD4+ T cells increasingly secreted IFN-γ and IL-17 whereas CD8+ T cells showed an enhanced secretion of IFN-γ and an increased mRNA expression of *CXCR6* and *CXCR3*. Respective effects of both CD4+ and CD8+ T cells could be abrogated by knockdown of NLRP3 or TRPM2 as well as blocking of IL-1β/IL1-R signaling in H_2_O_2_-treated HaCaT (75). These findings were corroborated in another study, which found CXCR3+ cell attracting CXCL9 chemokine significantly expressed in both lesional and perilesional skin of vitiligo patients compared to skin of healthy controls. Moreover, *CXCL9* mRNA expression was increased in NHEK in response to ATP-induced inflammasome activation mediated *via* ROS in comparison to untreated keratinocytes, indicating NLRP3 as corresponding inflammasome sensor (74). Released ATP led to activation of caspase-1, production of IL-1β and IL-18 *via* the P2X7 receptor in an inflammasome-dependent manner in keratinocytes and melanocytes as well as apoptotic cell death of the latter (74). Moreover, pharmacological inhibition of the P2X7 receptor significantly decreased ATP-dependent inflammasome activation, melanocyte apoptosis and CXCL9 release from keratinocytes (74).

### 4.4 Alopecia areata

AA represents an autoimmune disorder characterized by non-scarring hair loss ranging from small, limited lesions to complete loss of all body hair (93). AA manifests histologically with an inflammatory infiltrate composed predominantly of lymphocytes and Langerhans cells around the anagen hair bulb, which can lead to edema, recruitment of other infiltrating immune cells such as macrophages as well as apoptosis and necrosis (171, 172). AA is characterized by (i) an upregulation of NKG2D ligands, (ii) upregulation of MHC I and MHC II molecules, (iii) a decrease of local regulatory molecules such as transforming growth factor-β1, IL-10 and α-melanocyte-stimulating hormone and (iv) an increase of cytokines such as type I and type II IFNs, IFN-inducible chemokines (e.g. CXCL9, CXCL10, CXCL11), IL-15 and IL-2 (173). CXCL chemokines are known to recruit CXCR3+ effector T cells, which accumulate around hair bulbs and exhibit autoreactivity to follicular epithelial cells. These inflammatory events lead to loss of hair follicle immune privilege with anagen arrest (89–91, 100, 173, 174).

Regarding the inflammasome and associated proteins, early studies revealed a strong genetic association with both pro- and anti-inflammatory members of the IL-1 family with AA: In 1994, Tarlow et al. found polymorphisms in the IL-1 receptor antagonist gene (*IL1RN*) not only significantly associated with AA, but also disease severity, compared to healthy controls (131). These results were supported by AlFadhli et al., who found polymorphisms in *IL1B* and *IL1RN* significantly associated with the susceptibility to AA (132). The IL-1 receptor antagonist protein (IL-1Ra) competes for the receptor of IL-1α and IL-1β without giving rise to a proinflammatory signal, which led to the assumption that AA patients lacking a sufficient secretion of IL-1Ra may have a more progressive disease (175). *IL1B* mRNA expression was found to be significantly increased in scalp biopsies of AA patients compared to healthy controls and stimulation of human hair follicles with IL-1β and IL-1α *in vitro* resulted in an irreversible inhibition of hair growth (135–137, 176), which led to the hypothesis of IL-1 as a crucial mediator of hair growth arrest in AA. Moreover, SNPs in *IL18* on chromosome 11 (rs187238 (133, 134), rs1946518 (133), rs549908 (134)) are suggested to be a risk factor for AA susceptibility (133, 134) and serum concentrations of IL-18 in AA patients with extensive disease were found to be significantly higher than in healthy controls (139). In two large genome-wide association studies (GWAS) of AA, a number of polymorphisms were found in genes controlling innate and adaptive immune responses, such as T cell-associated genes (*IL-2RA, IL-2, IL4, IL13, CTLA4* and *ICOS*) and HLA genes (177, 178). However, in the respective GWAS, no polymorphisms associated with AA were found in genes of inflammasome components.

NLRP3, ASC, caspase-1 and IL-1β protein expression was found to be significantly increased in lesional skin of AA patients, particularly in the outer root sheath (ORS) of hair follicles and in infiltrating immune cells compared to scalp skin of healthy controls (138). Released nucleic acids were proposed as both primer and activator of the inflammasome as stimulation of AA patient derived cultured ORS cells with polyinosinic:polycytidylic acid (poly(I:C)), a synthetic RNA analog and well known TLR3 ligand, was found to induce (i) increased mRNA expression of inflammasome components *NLRP3, ASC* and *CASP1* and *IL1B*, (ii) enhanced protein expression of NLRP3, pro-caspase-1 and pro-IL-1β, (iv) increased secretion of active capsase-1 and IL-1β and (v) increased co-localization of NLRP3 and ASC compared to unstimulated cultured ORS cells (138). Moreover, knockdown of caspase-1 and NLRP3 *via* microRNA significantly inhibited poly(I:C)-induced IL-1β secretion, indicating that IL-1β secretion was dependent on inflammasome activation (138). Of note, NF-κB pathway was revealed to mediate poly(I:C) induced IL-1β secretion in cultured ORS cells (138).

### 4.5 Bullous pemphigoid

BP belongs to blistering autoimmune skin diseases and is characterized by the presence of specific autoantibodies directed against dermo-epidermal structural proteins such as hemidesmosome-targeting anti-BP160-NC16A antibody (102). In recent years, evidence for involvement of the NLRP3 inflammasome in BP pathogenesis has been found. Fang et al. found that in PBMCs from BP patients, the mRNA expression of NLRP3 inflammasome components *NLRP3*, *CASP1*, and *IL18* as well as the protein expression of NLRP3, ASC, pro-caspase-1, caspase-1 and pro-IL-18 were significantly increased compared to healthy controls (140). *AIM2* and *NLRP1* mRNA, however, were expressed significantly lower as compared to healthy controls. IL-1β mRNA or protein was not significantly increased in PBMCs compared to healthy controls and no obvious differences were observed in serum or blister fluid concerning IL-1β concentrations between BP patients and healthy controls. This contrasts with another study by Le Jan et al., who found IL-1β protein significantly increased in blister fluid of BP patients compared to healthy controls (18). Moreover, this group found IL-1β concentrations in blister fluid to positively correlate with the presence of erythema and urticarial plaques, hypothesizing that the inflammasome may play a role in the inflammatory phase preceding blister formation in BP and therefore might represent an early therapeutic target (18).

IL-18 concentrations were found to be increased in both serum blister fluid compared to healthy controls (140). Moreover, IL-18 protein was found to be strongly expressed particularly in the epidermis of lesional skin of BP patients compared to skin of healthy controls (140). Serum IL-18 concentrations also correlated positively with anti-BP180-NC16A autoantibody titers in serum of BP patients (140) which are also known to correlate positively with disease activity (179). Of note, after successful treatment of BP patients with traditional systemic corticosteroids, mRNA expression of *NLRP3, CASP1* and *IL18* in PBMCs as well as serum IL-18 concentrations were significantly reduced, leading to the hypothesis that the inflammasome contributes to disease pathogenesis (140).

In another study, NLRP3 protein was found to be expressed in the epidermis in keratinocytes, moreover in the dermis and blister cavity, particularly in skin resident cells such as endothelial cells and fibroblasts as well as infiltrating inflammatory cells such as macrophages and polymorphonuclear cells (18). It was shown that the downstream activated cytokines IL-23 and IL-17 could enhance expression of *IL1B* mRNA in monocyte-derived macrophages from BP patients (18). IL-17 also led to a significant increase in *NLRP3* mRNA expression in the corresponding macrophages (18). These results suggest that these cytokines may serve as “primers” and thus potentiate the effects of the inflammasome, resulting in a self-perpetuating inflammatory loop.

### 4.6 Psoriasis

Psoriasis is a chronic inflammatory skin disorder, which is characterized by a pathogenic mixture of both autoimmune and autoinflammatory immune responses. The most common subtype of this morphologically heterogenous disease, psoriasis vulgaris, manifests on the skin with demarcated plaques covered with silvery scales, often symmetrically on the scalp, elbows, knees and lower back (104). In addition, the disease can affect a variety of organ systems such as the joints in psoriatic arthritis or be accompanied by concomitant disorders such as cardiovascular disease or metabolic syndrome (104). Skin lesions are characterized by dysregulated keratinocyte differentiation with epidermal hyperproliferation, presenting histologically with loss of stratum granulosum and an immune cell infiltrate dominated by T cells in the dermis, which can be accompanied by epidermal clusters of neutrophils (180).

First evidence of a possible involvement of the inflammasome in psoriasis emerged more than twenty years ago, in which a dysregulation of IL-1 and increased protein expression of IL-18 in lesional skin of psoriasis patients compared to skin of healthy controls were detected (148, 181–183). SNPs in inflammasome-associated genes including *NLRP1* (rs8079034C and rs878329C) (141), *NLRP3* (rs3806265, rs10754557 and rs10733113) (142, 143), *AIM2* (rs2276405) (144), and *CARD8* (rs2043211) (142) have been associated with increased susceptibility to psoriasis in affected patients compared to healthy controls in a number of studies in different ethnic subgroups. Moreover, a single nucleotide variant (rs11652075) within the gene for the inflammasome regulatory protein and COP family member CARD14 leads to an increased NF-κB signaling and was found associated with disease development (145–147). Several other SNPs in *CARD14* have also been described to be associated with psoriatic skin disease (146).

The expression of inflammasome-associated proteins in the skin of psoriasis patients has been investigated in several studies so far. In psoriasis patients, mRNA of inflammasome-associated genes was found increased in lesional skin compared to non-lesional skin and skin of healthy controls, including ligand sensors *AIM2* (149), *IFI16* (149), *NLRP3* (150), *NLRP10* (149), inflammasome components *PYCARD* (encoding ASC protein) (149) and *CASP1* (150), the effector cytokines *IL1B* (149, 150) and *IL18* (148, 151) as well as regulatory *CARD18* (149). It was also shown that protein expression of NLRP3 (150), IFI16 (149), AIM2 (64), ASC (149), caspase-1 (64, 150), IL-1β (64, 150) and IL-18 (148) was increased in psoriatic skin lesions compared to the skin of healthy control subjects. Of note, Dombrowski et al. found that AIM2 protein was most intense expressed in the apical keratinocyte layers of psoriatic skin lesions (64), in contrast to de Koning et al., who found that AIM2 protein expression was most intense in the basal layer and adjacent to neutrophil-clusters in the stratum corneum (23). Surprisingly, pronounced NLRP10 protein expression was observed throughout the epidermis in both lesional psoriatic and healthy skin (149). Furthermore, mRNA of *NLRP1*, *NLRP3*, *AIM2*, *PYCARD* and *CASP1* was observed in both lesional and non-lesional epidermis of psoriasis patients (23, 152).

*In vitro*, AIM2 mRNA and protein was detected to be upregulated as a response to IFN-γ in human cultured keratinocytes (23, 64). When cultured keratinocytes were primed with IFN-γ and TNF-α, exposure to a synthetic DNA analog, poly(deoxyadenyl-deoxythymidyl) acid (poly(dA:dT)), significantly increased IL-1β release and secretion of bioactive caspase-1 (64). Knockdown of AIM2 was found to inhibit IL-1β release in response to poly(dA:dT) (64). The group further demonstrated that fragmented DNA was detectable in the cytosol of keratinocytes from psoriatic skin lesions compared to keratinocytes from healthy skin, although the source of DNA – whether it was excited from the nucleus or taken up from neighboring cells – was not identified (64). They also showed that LL37 promotes the uptake of DNA into keratinocytes by acting as a natural transfectant through complexation with DNA (64, 184). Surprisingly, simultaneous exposure of complexed LL37 and DNA resulted in significantly inhibited IL-1β release in cultured keratinocytes (64). Although incorporation of LL37-DNA complexes into other cells, e.g. pDCs, can achieve substantial proinflammatory effects (107), the formation of inflammasomes in keratinocytes was found abrogated by LL37-delivered cytosolic DNA (64), indicating LL37 as physiological inhibitor of AIM2-dependent inflammasome activation in the respective cells.

The negative regulatory molecule CARD18 revealed an increased protein expression in skin lesions of psoriasis patients, but particularly in non-lesional skin of psoriasis patients, compared to skin of healthy donors (153). In cultured keratinocytes pretreated with IFN-γ and TNF-α and stimulated with poly(dA:dT), silencing of *CARD18* with small interfering (si)RNA resulted in a significant decrease in *AIM2* and *CASP1* mRNA expression, however, affected an elevated IL-1β secretion (153), hypothesizing that, in psoriasis, inhibition of IL-1β by CARD18 may entail increased inflammasome priming *via* a regulatory feedback mechanism. The exact role of CARD18 in psoriasis remains to be elucidated as both CARD18 and IL-1β protein expression were found increased in lesional skin of psoriatic patients.

Another group found NLRC4 protein, a known ligand sensor capable of forming an inflammasome (185, 186), in the scale extract of patients with severe psoriatic eruptions, however, it was not detected in the scale extract of patients with mild psoriatic eruptions, with atopic dermatitis or of healthy controls (53). Accordingly, NLRC4 protein expression was increased in psoriatic lesional skin compared to non-lesional psoriatic skin and skin of healthy controls (53). The group also found that in keratinocytes cultured *in vitro*, *NLRC4* mRNA expression could be increased by several stimuli and conditions compared with growth-phase keratinocytes, including keratinocyte confluence, air exposure (for 48 hours), UV radiation, elevated Ca^2+^ levels, and oxidative stress from H_2_O_2_ exposure (53). Whether an NLRC4 inflammasome with corresponding effector functions is assembled in psoriasis skin lesions remains elusive.

The non-canonical inflammasome component *CASP5* was also found upregulated on mRNA level in lesional psoriatic skin compared to non-lesional skin of psoriasis patients and healthy skin (152). On protein level, however, only full-length and not bioactive caspase-5 was increased in lesional skin in comparison to healthy controls. Furthermore, *CASP5* mRNA was shown to be increased *in vitro* in response to stimulation of cultured keratinocytes with IFN-γ (152). A downregulation in *CASP5* mRNA expression was observed in the lesional skin of psoriasis patients treated with a TNF inhibitor compared to their skin lesions before the start of the respective therapy (152), indicating a prevention of TNF-induced inflammasome priming. However, since caspase-5 was not present in the bioactivated state and thus could not exhibit effector functions, the contribution of bioactive caspase-5 to homeostasis and disease remains to be studied.

Corresponding with findings of lesional skin, peripheral blood levels of ASC, IL-1β and IL-18 were found increased in untreated psoriasis patients compared to healthy controls (154, 155). *Ex vivo* peripheral blood CD4+ lymphocytes, CD14+ monocytes and CD16+ neutrophils revealed an increased protein expression of NLRP1 and NLRP3, in the latter two also of AIM2, compared to healthy controls (154). Exposure of whole blood to TNF-α resulted in a significant increase in *NLRP3*, *IL1B* and *IL18* mRNA expression compared to unstimulated samples (154). Stimulation of CD14+ and CD16+ peripheral blood cells with TNF-α, which is elevated in the plasma of psoriasis patients compared to healthy controls, also revealed an increase in caspase-1 activity (154), suggesting that TNF-α serves not only as a primer but also as a direct activator of the NLRP3 inflammasome. TNF-α signaling in monocytes was shown to proceed through the TNF receptor-caspase-8-caspase-1 pathway and did not result in elevated blood lactate dehydrogenase levels (154), indicating activation of the “alternative inflammasome” pathway without execution of pyroptosis. Accordingly, treatment with anti-TNF-α antibodies attenuated *IL1B* mRNA expression in whole blood compared to healthy control samples (154). These results support the recognition of psoriasis as systemic disease. Furthermore, it was concluded that ASC and IL-18 peripheral blood proteins can also be used clinically as inflammatory biomarkers for screening, disease prognosis and estimation of therapeutic response in psoriasis patients (155).

To date, several therapeutic agents specifically targeting the inflammasome have been investigated *in vitro* in cell culture disease models and *in vivo* in murine models with psoriasis-like phenotype, several of which show promising results (187).

## 5 Concluding remarks

In the last 20 years, knowledge of the inflammasome has revolutionized the field of innate immunity. Inflammasomes are being studied in a broad range of diseases, and specific inhibitors of the inflammasome are under development in preclinical and clinical trials (188–191). The inflammasome is also being intensively investigated in other inflammatory skin conditions such as atopic dermatitis, which is reviewed elsewhere (187). Aberrant inflammasome activation in autoimmune skin diseases, however, are still understudied. It needs to be determined more precisely which inflammasomes in which cell types are activated and whether they exert rather proinflammatory or pro-homeostatic functions. For instance, in mouse models of contact hypersensitivity (192) and cutaneous leishmaniasis (193) it was shown that NLRP10 can both promote proinflammatory or anti-inflammatory immune responses, dependent on the context. NLRP10 may be anti-inflammatory player as it can negatively regulate caspase-1-dependent IL-1β secretion and inhibit ASC‐mediated NF‐κB activation (194–196). Recently, NLRP10, which is also known to be highly expressed in the stratum granulosum in differentiated keratinocytes of human skin (27), was found to play a key role as an inflammasome in monitoring mitochondrial integrity by sensing mitochondrial damage in a DNA-independent manner in keratinocytes (25). The single nucleotide variants rs59039403 and rs878860 in *NLRP10* were found to be associated with atopic dermatitis in the Japanese population (197–200). It will be interesting to investigate under what circumstances ligand sensors such as NLRP10 form an inflammasome and exert either proinflammatory or anti-inflammatory functions in human skin. It is also of importance to understand the role of inflammasomes in terminal differentiation and their dysregulation in autoimmune skin diseases in more detail.

Furthermore, it is important to enlighten the complex interplay of regulatory mechanisms of inflammasomes and their dysregulation in disease. For instance, SNPs in *CARD14* can entail – as gain-of-function variant in psoriasis – increased NF-κB signaling and thus expression of proinflammatory cytokines (201). The loss-of-function variant in CARD14 in atopic dermatitis leads to a decrease in NF-κB activation, epidermal secretion of antimicrobial peptides (202) and mRNA expression of *FLG* (203), encoding filaggrin, which is an epidermal protein essential for skin barrier function (204). It needs to be investigated which regulatory proteins are expressed in autoimmune skin conditions and how they influence the inflammatory potential of inflammasomes in keratinocytes.

In addition, the cross-talk between NF-κB and antiviral IFN signaling pathways in the skin needs to be further elucidated. IFN signaling is critical in the majority of skin autoimmune diseases discussed. Its interaction with the inflammasome is a requirement for effective antiviral and antimicrobial host defence and tightly regulated (205, 206). In autoimmune diseases such as SLE (207) and Sjögren’s syndrome (208), IFN signaling can lead to increased expression and enhanced activation of the inflammasome and pyroptosis, thereby promoting disease progression.

These aspects are only examples of questions that should be addressed in further studies. Ideally, skin-specific roles of inflammasomes are assessed in a holistic manner, in which not only sensors of known potential are investigated but rather all inflammasomes are assessed simultaneously, in a cell-specific manner. Then, the inflammasome signatures can be determined that is required to maintain skin homeostasis, for example in non-inflammatory cell death of terminally differentiating keratinocytes, and compared the signature obtained from studying lesional skin. Such an inflammasome signature should be complemented with a GSDM signature and an IL-1 family signature.

Taken together, this review highlights that in the first two decades of inflammasome research, important evidence of aberrant inflammasome activation as a driving force of human autoimmune skin diseases has been reported. In several of the discussed diseases, inhibiting a specific type of inflammasome reduces inflammatory signals (e.g. CXCL chemokines) that otherwise drive the adaptive immune system, highlighting its relevance as potential therapeutic target. Notably, previous studies focused primarily on individual sensors for canonical inflammasomes formed after triggering of NLRP1, NLRP3 and AIM2, and examined individual regulatory mechanisms. In addition, it needs to be clarified whether inflammasomes exert either proinflammatory or pro-homeostatic effects in each respective context. We assume that a complete picture of inflammasome signaling, containing both its physiological role and its contribution to disease, will be of particular importance in identifying new therapeutic targets for the treatment of autoimmune skin diseases.

## Author contributions

TF drafted the manuscript and designed the tables and figure. DMDG contributed in writing, reviewing and editing of the manuscript. JW and IC revised the manuscript. All authors contributed to the article and approved the submitted version.
